# Impaired Brain Mitochondrial Bioenergetics in the Ts65Dn Mouse Model of Down Syndrome Is Restored by Neonatal Treatment with the Polyphenol 7,8-Dihydroxyflavone

**DOI:** 10.3390/antiox11010062

**Published:** 2021-12-28

**Authors:** Daniela Valenti, Fiorenza Stagni, Marco Emili, Sandra Guidi, Renata Bartesaghi, Rosa Anna Vacca

**Affiliations:** 1 Institute of Biomembranes, Bioenergetics and Molecular Biotechnologies (IBIOM), National Research Council (CNR), 70126 Bari, Italy; r.vacca@ibiom.cnr.it; 2 Department for Life Quality Studies, University of Bologna, 47921 Rimini, Italy; fiorenza.stagni2@unibo.it; 3 Department of Biomedical and Neuromotor Sciences, University of Bologna, 40126 Bologna, Italy; marco.emili2@unibo.it (M.E.); sandra.guidi3@unibo.it (S.G.)

**Keywords:** Down syndrome, Ts65Dn mice, brain mitochondria, oxidative phosphorylation, mitochondrial respiratory chain, 7,8-dihydroxyflavone

## Abstract

Down syndrome (DS), a major genetic cause of intellectual disability, is characterized by numerous neurodevelopmental defects. Previous in vitro studies highlighted a relationship between bioenergetic dysfunction and reduced neurogenesis in progenitor cells from the Ts65Dn mouse model of DS, suggesting a critical role of mitochondrial dysfunction in neurodevelopmental alterations in DS. Recent in vivo studies in Ts65Dn mice showed that neonatal supplementation (Days P3–P15) with the polyphenol 7,8-dihydroxyflavone (7,8-DHF) fully restored hippocampal neurogenesis. The current study was aimed to establish whether brain mitochondrial bioenergetic defects are already present in Ts65Dn pups and whether early treatment with 7,8-DHF positively impacts on mitochondrial function. In the brain and cerebellum of P3 and P15 Ts65Dn pups we found a strong impairment in the oxidative phosphorylation apparatus, resulting in a deficit in mitochondrial ATP production and ATP content. Administration of 7,8-DHF (dose: 5 mg/kg/day) during Days P3–P15 fully restored bioenergetic dysfunction in Ts65Dn mice, reduced the levels of oxygen radicals and reinstated the hippocampal levels of PGC-1α. No pharmacotherapy is available for DS. From current findings, 7,8-DHF emerges as a treatment with a good translational potential for improving mitochondrial bioenergetics and, thus, mitochondria-linked neurodevelopmental alterations in DS.

## 1. Introduction

Triplication of human chromosome 21 (Hsa21) causes Down syndrome (DS), which represents the most frequent genetic abnormality leading to intellectual disability. DS presents neuropathological phenotypes that are already detectable during foetal life and infancy and bring about alterations in brain development [1]. The brains from subjects with DS show structural and functional abnormalities, including reduced volume, lower neuronal density and abnormal synaptic plasticity [2]. Multiple and complex molecular mechanisms trigger brain development alterations in DS and there are currently no therapies to recover the neurocognitive impairment occurring in DS.

During embryonic development, neural stem cells proliferate and differentiate into neurons in a process called neurogenesis [3]. Notably, in the hippocampus, neurogenesis is not limited to the prenatal period, but continues throughout life as an adaptive response regulating brain plasticity and memory [4]. Reduced neurogenesis is one of the major neurodevelopmental defects leading to cognitive disability in DS [5]. Results obtained using different model systems, including brain biopsies from DS subjects, DS mouse models and neural progenitor cells (NPCs) derived from the hippocampus of mouse models of DS, showed a reduction in hippocampal proliferation potency, impaired neuronal maturation and reduced connectivity in the neonatal period. This stage of life, together with the prenatal period, represents a critical window for therapeutic interventions aimed at improving the neurodevelopmental alterations of DS [6,7,8,9].

Neuronal developmental processes, including cell proliferation and differentiation, axonal and dendritic growth, and formation of dendritic spines, are strongly dependent on the energy produced by brain mitochondria through oxidative phosphorylation (OXPHOS) [10]. Indeed, defects in energy metabolism negatively compromise neuronal development, survival and function [11] and represent early events in several neurodevelopmental intellectual disability-linked diseases [12,13,14], including DS (for reviews, see [15,16,17]).

Several in vitro studies from our and other research groups have shown alterations in the mitochondrial structure, bioenergetics and biogenesis associated with unbalanced redox homeostasis in human peripheral cell lines with Hsa21 trisomy, such as foetal fibroblasts and lymphoblastoid cells [18,19,20,21]. In cultured NPCs isolated from the hippocampus of the Ts65Dn mouse model of DS, we also highlighted a causal correlation between bioenergetic dysfunction and reduced in vitro neurogenesis, both reversed by the polyphenols resveratrol and epigallocatechin 3-gallate (EGCG) [22,23].

In a pilot study in young children with DS aged 1 to 8 years, we have recently explored the safety profiles and the efficacy of EGCG in restoring the deficit of mitochondrial complex I and V activities [24].

Polyphenols are phytochemical bioactive compounds known for exerting numerous beneficial effects in the brain, both in health and disease [25,26,27]. In the literature, the capability of polyphenols to activate mitochondria through modulation of the signalling pathways regulating mitochondrial functions has been proven (for references, see [28]). They also modulate the activity and the expression of the key Hsa21 genes involved in DS pathogenesis, such as dual specificity tyrosine-phosphorylation-regulated kinase 1A (DYRK1A), regulator of calcineurin 1 (RCAN1), amyloid precursor protein (APP) and some miRNAs, such as the miR-155, as well as no-Hsa21-encoded key regulatory proteins for brain development, such as the tropomyosin-related kinase B (TrkB) and the brain-derived neurotrophic factor (BDNF) [29]. Therefore, polyphenols have been proposed as potential phyto-drugs for the management of some DS-associated clinical manifestations (for references, see [15,16,28]), as well as for prenatal intervention [30]. Indeed, some of them have been tested for their ability to activate neurogenesis in vivo in DS mouse models [31,32,33,34]. In particular, the flavonoid 7,8-dihydroxyflavone (7,8-DHF), which exerts important positive effects in numerous mouse models of brains disorders, including DS [29], when supplied to Ts65Dn mice during neonatal life (postnatal days P3-P15), fully restored hippocampal neurogenesis, the total number of granule neurons and dendritic spine density [33].

In vivo preclinical data on brain mitochondrial bioenergetics, which represents the main source of ATP in the central nervous system during development and neurogenesis [35], are still lacking in DS. Moreover, the effects of the flavonoid 7,8-DHF on mitochondrial function in DS remain to be elucidated. To fill this gap, in the current study we sought to establish whether mitochondrial bioenergetics is already compromised during neonatal life in Ts65Dn mice, a widely used model of DS, and explored the possibility that the polyphenol 7,8-DHF is capable of modulating mitochondrial OXPHOS.

## 2. Materials and Methods

### 2.1. Experimental Subjects and Treatment

Ts65Dn mice were obtained by crossing B6EiC3Sn a/A-Ts(17^16) 65Dn females (JAX line 1924) with C57BL/6JEiJ × C3H/HeSnJ (B6EiC3Sn) F1 hybrid males (JAX line 1875; euploid males). These mice were supplied by Jackson Laboratories (Bar Harbor, ME, USA). We used here mice of the first breeding generation, in order to maintain the original genetic background. Genotyping was carried out as previously described [33]. The day of birth was considered as postnatal (P) day zero (P0). The animals’ health and well-being were checked by the veterinary service. The animals were kept in a room with a 12:12 h light/dark cycle. Water and food were available ad libitum.

Experiments were carried out in compliance with the European Communities Council Directive of 24 November 1986 (86/609/EEC) for the use of experimental animals and after approval by Italian Ministry of Public Health (205/2019-PR). In this study, we sought to minimize animal discomfort and the number of used animals.

All pups that survived during the period from P0 to P3 entered this study. A first cohort of P3 pups of either sex (euploid mice: *n* = 5; Ts65Dn mice: *n* = 5) was used for evaluation of mitochondrial function. A second cohort of mice of either sex were daily subcutaneously injected with 7,8-DHF (5.0 mg/kg dissolved in vehicle) or the vehicle (PBS with 1% DMSO) from P3 to P15 and were used for evaluation of the effect of treatment with 7,8-DHF on mitochondrial function (euploid mice treated with vehicle: *n* = 5; Ts65Dn mice treated with vehicle: *n* = 5; euploid mice treated with 7,8-DHF: *n* = 5; Ts65Dn mice treated with 7,8-DHF: *n* = 5) and for Western blotting analysis (euploid mice treated with vehicle: *n* = 9; Ts65Dn mice treated with vehicle: *n* = 8; euploid mice treated with 7,8-DHF: *n* = 5; Ts65Dn mice treated with 7,8-DHF: *n* = 7). The dose of 5.0 mg/kg of 7,8-DHF was chosen because it is able to fully restore hippocampal neurogenesis [33].

### 2.2. Brain Tissue Dissection and Mitochondria Isolation

The animals were sacrificed, their brains were dissected, and brain hemispheres and cerebella were cryopreserved and stored at −80 °C until assayed, as described in [36].

### 2.3. Measurements of Mitochondrial ATP Production

The rate of OXPHOS-dependent ATP synthesis was measured, as previously described [13], in mitochondria isolated from both hemispheres and cerebella (0.5–0.3 mg protein, respectively).

### 2.4. Measurement of Mouse Brain ATP Levels

Perchloric acid extracts from P3 and P15 mouse brain hemispheres (approx. 70 and 150 mg, respectively) were obtained as reported in [13]. ATP tissue level was measured as displayed in [17].

### 2.5. Measurement of Mitochondrial Respiratory Chain Complex (MRC) Activities

Measurements of MRC activities were performed by isolating mitochondrial membrane-enriched fractions from brain mitochondria as in [13]. The activity assays were carried out, as previously described [13], by performing the three following sequential measurements: (i) rotenone-sensitive complex I (as NADH decylubiquinone reductase activity) followed by the oligomycin-sensitive ATPase activity of complex V; (ii) malonate-sensitive complex II activity; and (iii) the activity of complex IV trigged by the reduced cytochrome c followed by the antimycin-sensitive complex III.

### 2.6. Detection of Reactive Oxygen Species (ROS) Levels

ROS levels were detected by incubating the probe 2′,7′-dichlorofluorescin diacetate (DCFH-DA, 20 μM) for 30 min at 37 °C, with the supernatants (200 μL) obtained from the centrifugation of homogenates of mouse brain hemispheres (5500× *g* for 3 min). The fluorescence of DCF (λex-em = 488–530 nm), deriving from DCFH-DA oxidation, was expressed as arbitrary fluorescence units/mg of sample protein and then converted into % of untreated euploid values, as in [14].

### 2.7. Western Blotting

Total proteins were extracted from hippocampal homogenates of P15 mice, obtained as previously described [37], and the levels of the following proteins were quantified: PGC1-α (primary antibody: rabbit polyclonal 1:1000, Millipore, ab3242; secondary antibody: HRP-conjugated anti-rabbit 1:10,000, Jackson Immunoresearch, 111-035-003) and α-Tubulin (primary antibody: mouse monoclonal 1:1000, Sigma-Aldrich, Milano, Italy, Clone B-5-1-2, T5168; secondary antibody: HRP-conjugated anti-mouse 1:10,000, Jackson Immunoresearch, 115-035-003). We carried out densitometric analysis of the digitized images with a ChemiDoc XRS+ system equipped with Image Lab software (Bio-Rad Laboratories, Hercules, CA, USA). The intensity of each band was expressed as the ratio of the intensity of the corresponding α-Tubulin band.

### 2.8. Statistical Analysis

Data are reported as the mean ± standard error of the mean (SE). Data were analysed with IBM SPSS 22.0 software. For each measure, we first checked the data distribution, using the Shapiro–Wilk test, and homogeneity of variance, using Levene’s test. If the data were normally distributed and the variance was homogeneous, statistical analysis was then carried out using either a one-way ANOVA or a two-way ANOVA with genotype (euploid or Ts65Dn genotype) and treatment (treatment with vehicle or treatment with 7,8-DHF) as factors. Subsequently, post-hoc multiple comparisons were performed with Tukey’s test. In the case that data were not normally distributed and the variance was not homogeneous, data were transformed to achieve normality. Based on the “Box plot” tool of SPSS Descriptive Statistics, from each analysis, the extremes, i.e., values that were larger than 3 times the IQ range x ≥ Q3 + 3 × (IQ); x ≤ Q1 − 3 × (IQ), were excluded. The legends of figures report the number of mice included in (and excluded from, if any) individual analyses. A probability level of *p* ≤ 0.05 was considered to be statistically significant.

## 3. Results

### 3.1. Brain Mitochondrial Bioenergetics in Ts65Dn Pups

**Figure 1:**
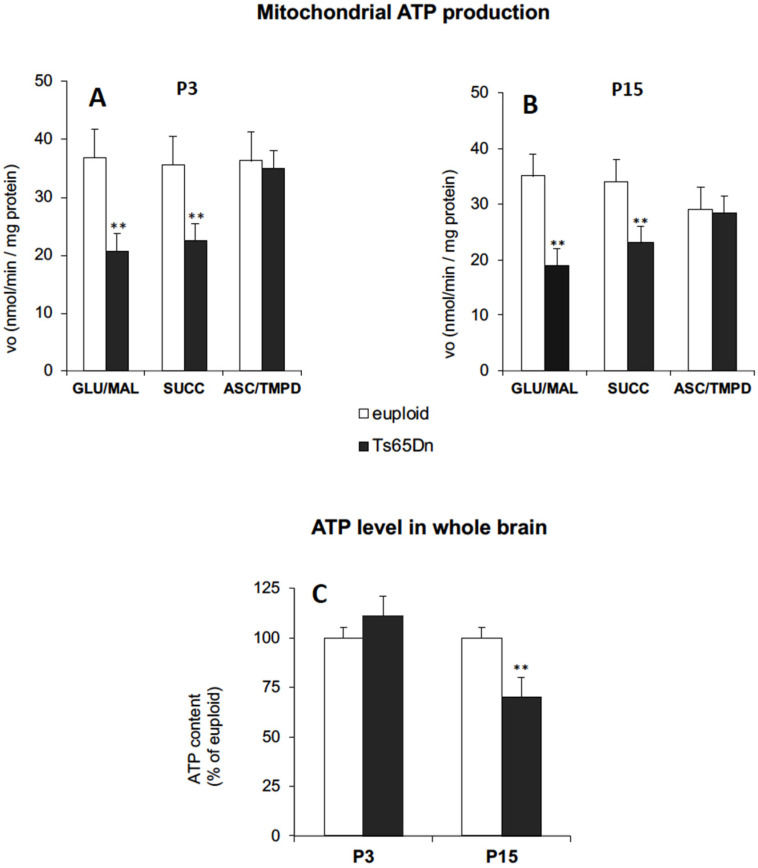
Aberrant mitochondrial bioenergetics in the Ts65Dn mouse brain. (**A**,**B**) The rate of mitochondrial ATP production (vo) was measured in mitochondria isolated from the brain hemispheres of P3 (**A**) and P15 (**B**) Ts65Dn mice and euploid mice, in the presence of the respiratory substrates glutamate plus malate (GLU/MAL; 5 mM each), succinate (SUCC; 5 mM) or ascorbate plus TMPD (ASC/TMPD; 5 mM and 0.5 mM, respectively), and expressed as nmoles ATP produced/min/mg protein. (**C**) ATP levels were assayed spectrophotometrically in neutralized perchloric acid extracts of P3 and P15 cryopreserved brain hemispheres of Ts65Dn and euploid mice and are expressed as % of euploid controls. Data are the mean ± SD of two independent measurements (*n* = 5 mice for each experimental group). Group differences were evaluated with Tukey’s test. ** *p* < 0.01: Ts65Dn vs. euploid counterparts.

To establish whether mitochondrial bioenergetics is already compromised during neonatal life stages in the Ts65Dn mouse model of DS, we first carried out a functional analysis of the mitochondrial bioenergetic efficiency in the brain of neonate mice. For this purpose, we measured the ATP production via OXPHOS in mitochondria isolated from cryopreserved brain hemispheres from Ts65Dn mice aged 3 (P3) and 15 (P15) days, compared with age-matched euploid mice (Figure 1).

To better dissect the efficiency of the OXPHOS process and disclose the relative contribution of each mitochondrial respiratory chain (MRC) complex composing OXPHOS machinery, the mitochondrial ATP production was evaluated by supplying the respiratory substrates of either complex I, glutamate plus malate (GLU/MAL), complex II, succinate (SUCC), or complex IV, ascorbate plus N,N,N′,N′-tetramethyl-p-phenylenediamine (ASC/TMPD).

The rate of mitochondrial ATP synthesis was strongly reduced in brain mitochondria from both P3 (Figure 1A) and P15 (Figure 1B) Ts65Dn mice compared to their euploid controls, when either GLU/MAL or SUCC were used as the energy substrates. Conversely, no significant differences between Ts65Dn and euploid mice were found in complex IV-dependent mitochondrial ATP production, regardless of neonatal age. Interestingly, the level of ATP measured in the brain hemispheres was significantly lower in P15 Ts65Dn mice, compared to the euploid controls (Figure 1C), but was not significantly decreased in the brain of P3 Ts65Dn mice, which is suggestive of a tentative compensatory event for energy deficit at very early life stages.

**Figure 2:**
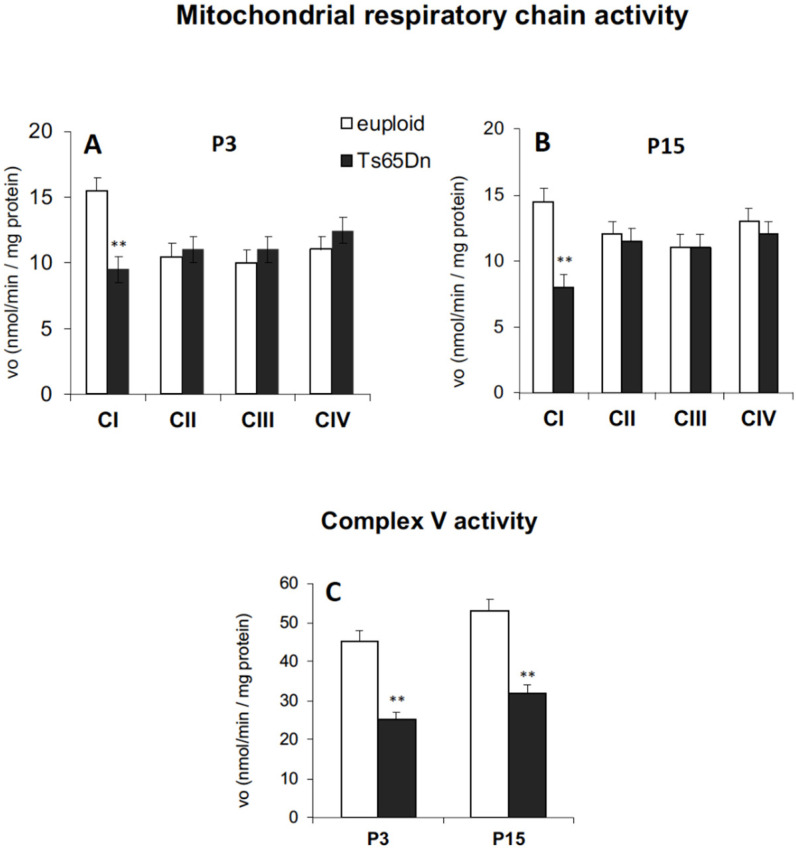
Reduced catalytic efficiency of MRC complex I and ATP synthase in Ts65Dn mouse brain. The activities of complex I, complex II, complex III, complex IV, (**A**,**B**) and complex V (**C**) were measured, as described in the Materials and Methods, in mitochondrial membrane-enriched fractions from cryopreserved P3 and P15 brain hemispheres of Ts65Dn mice and euploid mice. Rate values are the mean ± SD obtained in two independent measurements (*n* = 4 mice for each experimental group). Group differences were evaluated with Tukey’s test. ** *p* < 0.01: Ts65Dn vs. euploid counterparts.

In accordance with the results obtained by monitoring mitochondrial ATP production by OXPHOS, and in line with the dysregulated MRC complex levels found in 1–9-month-old Ts65Dn mice [38], functional analyses of all five MRC complexes revealed a significant reduction in the activities of complex I (Figure 2A,B) and ATP synthase (complex V) (Figure 2C) in both P3 and P15 Ts65Dn mice compared to their euploid controls.

### 3.2. Effect of Early Treatment with 7,8-DHF on Mitochondrial Bioenergetics

**Figure 3:**
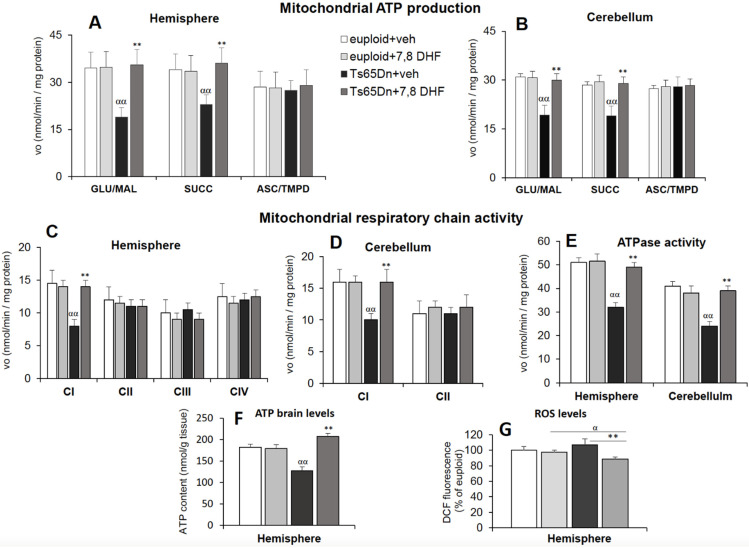
Neonatal treatment with 7,8-DHF improves the bioenergetic status in Ts65Dn mice. (**A**,**B**) Rate of ATP production was measured spectrophotometrically, as described in Figure 1, in isolated mitochondria from cryopreserved brain hemispheres (**A**) and cerebella (**B**) in the presence of the MRC respiratory substrates: glutamate plus malate (GLU/MAL), succinate (SUCC) or ascorbate plus TMPD (ASC/TMPD). Values are the mean ± SD of two independent measurements in 4 mice for each experimental group: vehicle-injected Ts65Dn mice (Ts65Dn + veh), 7,8-DHF-injected Ts65Dn mice (Ts65Dn + 7,8-DHF), vehicle-injected euploid mice (euploid + veh) and 7,8-DHF-injected euploid mice (euploid + 7,8-DHF). (**C**–**E**) Activity assays of complex I (CI), complex II (CII), complex III (CIII), complex IV (CIV) (**C**,**D**) and complex V (ATPase, CV) (**E**) were performed in mitochondrial membrane-enriched fractions from cryopreserved brain hemispheres and cerebella of vehicle-injected Ts65Dn mice (Ts65Dn + veh), 7,8-DHF-injected Ts65Dn mice (Ts65Dn + veh), vehicle-injected euploid mice (euploid + veh) and 7,8-DHF-injected euploid mice (euploid + 7,8-DHF). Rate values are the mean ± SD of two independent measurements in 4 mice for each experimental group, expressed as nmoles/min/mg protein. (**F**) Measurements of ATP brain levels were performed spectrofluorimetrically, as described in the Materials and Methods, in neutralized perchloric acid extracts of cryopreserved brain hemispheres of vehicle-injected Ts65Dn mice (Ts65Dn + veh), 7,8-DHF-injected Ts65Dn mice (Ts65Dn + veh), vehicle-injected euploid mice (euploid + veh) and 7,8-DHF-injected euploid mice (euploid + 7,8-DHF). Data are the mean ± SD of two independent measurements in 5 mice for each experimental group and expressed as nmoles ATP/g brain tissue. Significant differences between groups were calculated with one-way ANOVA and Tukey’s test. αα *p* < 0.01 Ts65Dn + veh vs euploid + veh; ** *p* < 0.01: Ts65Dn + 7,8-DHF vs Ts65Dn + veh vehicle. (**G**) ROS levels were detected as described in the Materials and Methods in cerebral hemisphere homogenates of vehicle-injected Ts65Dn mice (Ts65Dn + veh), 7,8-DHF-injected Ts65Dn mice (Ts65Dn + veh), vehicle-injected euploid mice (euploid + veh) and 7,8-DHF-injected euploid mice. Data are reported as the mean ± SD of 4 mice for each experimental group. ** = *p* < 0.01 Ts65Dn + 7,8-DHF vs. Ts65Dn + veh; α = *p* < 0.05 Ts65Dn + 7,8-DHF vs. euploid + veh by ANOVA with a post-hoc Turkey’s test.

Hippocampal neurogenesis and dendritic spine growth are strictly dependent on mitochondrial energy [10,39]. Given the proven efficacy of neonatal treatment with 7,8-DHF in restoring both these processes [33], we evaluated the effect of 7,8-DHF on mitochondrial energy efficiency. For this purpose, we monitored ATP production via OXPHOS in mitochondria isolated from the hemispheres of Ts65Dn and euploid mice aged 15 days subcutaneously administered with either 7,8-DHF (5 mg/kg/day) or the vehicle, during postnatal period P3–P15. We found that, in Ts65Dn mice, treatment with 7,8-DHF triggered complete recovery in the rate of complex I- and II-dependent mitochondrial ATP synthesis (Figure 3A).

The cerebellum belongs to the brain regions whose development is severely compromised in DS [40,41]. Therefore, we sought to establish whether early mitochondrial energy deficits are also present in this region and whether treatment with 7,8-DHF impacts on cerebellar mitochondrial function. In P15 Ts65Dn mice we found defects in mitochondrial bioenergetics similar to those observed in the cerebral hemispheres, suggesting widespread impairment of mitochondrial function in the developing brain (Figure 3B,D,E). Importantly, treatment with 7,8-DHF caused the rescue of mitochondrial energy deficit to values comparable to euploid mice also in mitochondria isolated from the cerebellum of Ts65Dn mice (Figure 3B). Treatment with 7,8-DHF fully rescued the activity of the defective MRC complex I (Figure 3C,D) and ATP synthase activity (Figure 3E) in both hemispheres and cerebella of Ts65Dn mice and increased the ATP brain levels to euploid values (Figure 3F).

Mitochondria are the main cell producers of ROS [42,43] and their defective function could contribute to the alteration of redox homeostasis and oxidative stress already reported in DS. Here, we assessed ROS levels in the hemispheres of P15 euploid and Ts65Dn mice and the effect of neonatal treatment with 7,8-DHF (Figure 3G). Although we found no significant difference in the basal ROS levels, regardless of their origin, between euploid and Ts65Dn mice, the ROS content underwent a significant reduction in treated Ts65Dn mice compared to both Ts65Dn and euploid mice treated with the vehicle. In contrast, treatment with 7,8-DHF did not change the baseline ROS levels in euploid mice, thus suggesting a greater antioxidant response to 7,8-DHF in Ts65Dn mice.

### 3.3. Effect of Early Treatment with 7,8-DHF on PGC-1α Levels

**Figure 4:**
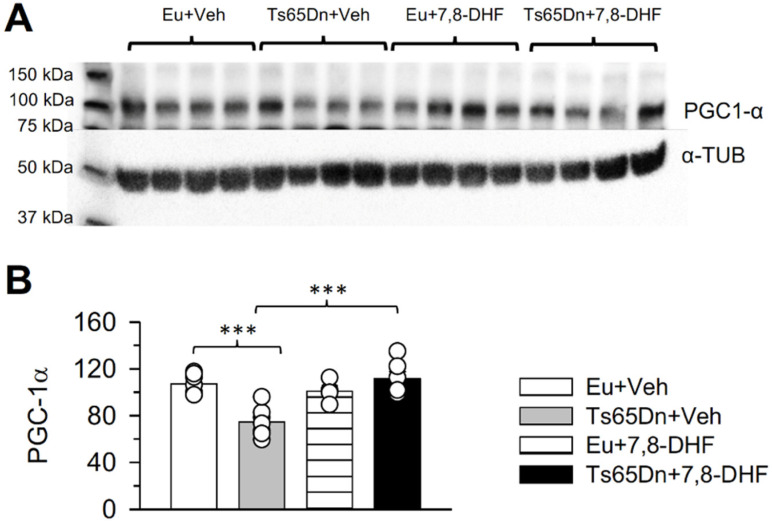
Outcome of treatment with 7,8-DHF in the neonatal period on PGC-1α expression in the hippocampal formation of Ts65Dn and euploid mice aged 15 days. Western blot analysis of PGC-1α expression in the hippocampus of P15 Ts65Dn and euploid mice treated either with vehicle (untreated mice) or 7,8-DHF (treated mice) during the postnatal days P3–P15. (**A**) Representative western blot images showing immunoreactivity for PGC-1α and the housekeeping gene α-Tubulin. (**B**) Levels of PGC-1α in the four experimental groups (untreated euploid and Ts65Dn mice and treated euploid and Ts65Dn mice). Values were normalized to α-Tubulin. Protein levels (mean ± SE) are expressed as a percentage of the values of untreated euploid mice. The scatterplots over each column represent the values of individual cases for each group. The following number of mice were analysed. Untreated euploid mice: *n* = 9; untreated Ts65Dn mice: *n* = 8; 7,8-DHF-treated euploid mice: *n* = 5; 7,8-DHF-treated Ts65Dn mice: n = 7. Two untreated euploid mice (yielding n = 7), one untreated Ts65Dn mouse (yielding *n* = 7), one 7,8-DHF-treated euploid mouse (yielding *n* = 4) and one 7,8-DHF-treated Ts65Dn mouse (yielding *n* = 6) were excluded from analysis based on the SPSS Box plot tool, as described in the Statistics section. *** *p* < 0.001 (Tukey’s test after two-way ANOVA). Abbreviations: 7,8-DHF, 7,8-dihydroxyflavone; α-Tub, α-Tubulin; Eu, euploid; Veh, vehicle.

The gene peroxisome proliferator activated receptor gamma coactivator 1 alpha (PGC-1α or PPARGC1A) is a key modulator of mitochondrial biogenesis and bioenergetics [44,45]. Various Hsa21 genes negatively regulate PGC-1α expression and function (see [17,46]), which may account for a reduction in mitochondrial biogenesis and OXPHOS defects. Previous evidence showed that the PGC-1α levels are reduced in hippocampal NPCs derived from the Ts65Dn mouse [22], suggesting that this defect is an important determinant of abnormal mitochondrial biogenesis and bioenergetics. Therefore, we sought to establish whether the beneficial effects of treatment with 7,8-DHF on the mitochondrial function of Ts65Dn mice were associated with changes in the PGC-1α levels. We found that in untreated Ts65Dn mice aged 15 days the hippocampal levels of PGC-1α were reduced in comparison with those of euploid mice (Figure 4B). In Ts65Dn mice treated with 7,8-DHF from P3 to P15 the levels of PGC-1α became significantly higher in comparison with those of untreated Ts65Dn mice and similar to those of vehicle-treated euploid mice (Figure 4B). In euploid mice, treatment had no effect on PGC-1α levels. These results suggest that the beneficial effect of treatment with 7,8-DHF on the mitochondrial function of Ts65Dn mice are mechanistically linked to an increase in PGC-1α levels.

## 4. Discussion

### 4.1. Early Alterations in Mitochondrial Function in Ts65Dn Mice

Our study provides novel evidence that the brain of neonate Ts65Dn mice exhibits defective functional activities of MRC complexes, i.e., complex I and ATP synthase, similar to the fibroblasts and lymphoblastoid cells from DS subjects [17,18,19] as well as Ts65Dn-derived hippocampal NPCs [21,22] and brain cortex of Ts16 mice [47]. This confirms that the Ts65Dn mouse, the first viable trisomy model [48], is a valid model for DS because it exhibits the mitochondrial phenotypic features of the syndrome very early during brain development, similarly to the DS condition. The level of ATP measured in the mouse brain was significantly lower in P15 mice, compared to euploid controls, thus suggesting that the inefficient mitochondrial ATP production found in Ts65Dn mice is an early event that translates into an alteration of the whole brain energy status. Interestingly, the mitochondrial energy deficit in P3 Ts65Dn mice was not associated with a decrease in the brain ATP content, suggesting that tentative compensatory events might occur early, such as an enhancement of glycolysis, as detected in foetal human Hsa21 fibroblasts [17].

Oxidative stress linked to mitochondrial deficits and an increase in the production of ROS have been previously detected in different DS-derived primary cells, including human foetal and adult fibroblasts [17,18,49], cortical neurons and astrocytes [50]. In particular, complex I was identified as a key determinant of alterations in ROS homeostasis in Ts16 neurons [51,52]. Accordingly, we previously demonstrated that the reduced catalytic activity of complex I is the primary cause for increased mitochondrial ROS in DS human peripheral cells [18,49].

The current finding that the brain hemispheres of neonate Ts65Dn mice exhibit no change in ROS level fits with similar evidence in NPCs from the Ts65Dn hippocampus [21] but is in contrast with data from human skin Hsa21 fibroblasts [18], although all these models exhibit MRC complex I impairment. These observations strongly suggest, firstly, that dysfunction of MRC complexes is an inherent feature of DS and not a consequence of ROS overproduction and, secondly, that ROS-preserving mechanisms might be activated in the DS brain, although the latter issue remains to be investigated.

### 4.2. Early Treatment with 7,8-DHF Restores Brain Mitochondrial Bioenergetics

This study demonstrates that the compromised and inefficient brain mitochondrial bioenergetics in neonate Ts65Dn mice can be rescued by neonatal treatment with the polyphenol 7,8-DHF. Recent data support an action of 7,8-DHF on mitochondrial dynamics in the brain and other tissues. In particular, 7,8-DHF reduces the gain in body weight, through an enhancement of mitochondrial biogenesis in skeletal muscle [53], inhibits doxorubicin-induced cardiotoxicity via enhancing mitochondrial oxidative phosphorylation [54], protects against ischemic cardiac damage through a selective action on mitochondrial dynamics [55], protects the brain from traumatic injury by normalizing the levels of molecules involved in mitochondrial biogenesis [56], and ameliorates hippocampal mitochondrial function and biogenesis in a model of HIV-associated neurocognitive disorder [57].

The flavonoids EGCG and resveratrol have been shown to counteract defective bioenergetics and biogenesis of mitochondria in cultures of NPCs derived from the hippocampus of the Ts65Dn mouse [22]. The current study shows that Ts65Dn pups treated with the flavonoid 7,8-DHF underwent restoration of brain mitochondrial bioenergetics. This knowledge expands the gamut of flavonoids that could be used as potential mitoprotective therapy for DS. The choice of the optimum therapy could be based on a comparative analysis of the bioavailability, extent and duration of the effects in the brain and other organs. It seems worthwhile to mention the fact that we tested here the effects of 7,8-DHF in vivo. Although systems in culture are very useful in view of their simplified condition, it cannot be taken for granted that the effects observed in vitro are replicated in vivo. The current study shows that Ts65Dn pups treated with the flavonoid 7,8-DHF underwent restoration of brain mitochondrial bioenergetics, indicating an effect on mitochondrial function in the in vivo condition.

Since neurons have a limited glycolytic capacity, approximately 10% only of their ATP is produced through glycolysis [58]. Therefore, mitochondrial bioenergetics is crucial for ATP-dependent processes that enable neurons to function and respond adaptively to environmental challenges. The current study shows that neonatal treatment with 7,8-DHF restores mitochondrial function in the brain of Ts65Dn mice. The process of brain maturation largely takes place in the period that spans from birth to puberty. This implies the necessity of an adequate metabolic rate in order to satisfy the demands inherent to maturation processes. We previously found that treatment with 7,8-DHF from P3 to P15 restored neurogenesis impairment and dendritic spine development in Ts65Dn mice [33]. The current finding that treatment with 7,8-DHF during this time window restores brain mitochondrial function suggests that a treatment-induced improvement in the metabolic rate may play an important contribution to these effects. Importantly, administration of 7,8-DHF during foetal and neonatal life stages does not cause adverse effects on the general growth of Ts65Dn mice [33,59], suggesting the safety of this flavonoid even at early life stages. Thus, 7,8-DHF may represent a possible therapeutic tool for improving mitochondrial bioenergetic efficiency, thereby supporting brain development in children with DS.

### 4.3. Early Treatment with 7,8-DHF Restores PGC-1α Levels in Ts65Dn Mice

Neuronal development and differentiation are largely modulated by the brain derived neurotrophic factor (BDNF). Its binding to its cognate receptor, the tropomyosin-related kinase receptor B (TrkB), triggers activation of several signalling events, including the phosphoinositide 3-kinase (PI3K)/Akt, Ras/extracellular signal-regulated kinase (ERK) and phospholipase C(PLCγ)/protein kinase C (PKC) pathways [29]. BDNF is down expressed in the brains of Hsa21 foetuses and Ts65Dn mice [60,61], which suggests substantial contribution of BDNF deficiency in DS-linked brain developmental alterations. The flavonoid 7,8-DHF is considered a BDNF mimetic because it is known to specifically bind to the TrkB receptor, mimicking the effects of BDNF, its natural ligand [27]. Thus, the effects of 7,8-DHF on DS-linked developmental alterations may be attributed to activation of TrkB signalling.

Current results showed that treatment with 7,8 DHF increased the levels of PGC-1α in Ts65Dn mice. Regarding the modulation of PGC-1α expression, it has been shown that it can be induced by metabolic challenges, such as exercise, ROS and the cyclic AMP response element binding protein (CREB) [58]. There is evidence that BDNF enhances PGC-1α expression and mitochondrial biogenesis through the activation of mitogen-activated protein kinases (MAPKs) and CREB [62]. The binding of 7,8-DHF to the TrkB receptor activates, similarly to BDNF, various intracellular signalling pathways, including the RAS–ERK–pCREB pathway [29]. We previously found that administration of 7,8-DHF in the time window P3-P15 increased the phosphorylation of ERK1/2 in Ts65Dn mice [33]; here, we found that 7,8-DHF can act as a natural activator of PGC-1α and is able to counteract mitochondrial energy dysfunctions. Taken together these results suggest that treatment with 7,8-DHF increases the PGC-1α levels through the RAS–ERK–pCREB pathway, thereby ameliorating mitochondrial activity. This conclusion is in line with evidence that activation of TrkB by its agonist 7,8-DHF initiates the CREB/PGC-1α cascade to provoke mitochondrial biogenesis and cellular respiration in skeletal muscle [53]. However, further mechanisms responsible for the effect of 7,8-DHF on restoration of brain mitochondrial bioenergetics, including an activation of mitophagy, mitochondrial remodelling and a consequent activation of mitochondrial biogenesis [63], cannot be excluded and merit to be deeply investigated.

## 5. Conclusions

Our results suggest that early treatment with 7,8-DHF may represent a therapeutic strategy with a good translational potential for improving the mitochondrial bioenergetic deficit that represents an inherent condition associated with neurodevelopmental alterations in DS. Likewise, we think that any therapeutic treatment aimed at improving mitochondrial energy efficiency should be encouraged and started at early life stages in order to prevent or at least mitigate the neurological alterations of DS. Prolonged treatment with polyphenols is likely to be necessary for DS, given the loss in adulthood of the short-time effects of neonatal treatment [29,61]. Yet, natural bioactive compounds, such as polyphenols, due to their multi-target action and the long-time safe profile [28], remain to be considered with interest as therapeutic interventions in DS and, possibly, other neurodevelopmental disorders.

## Data Availability

The data that support the findings of this study are available from the corresponding authors upon reasonable request.

## References

[1] Kazemi M., Salehi M., Kheirollahi M. (2016). Down syndrome: Current status, challenges and future perspectives. Int. J. Mol. Cell. Med..

[2] Rachidi M., Lopes C., Clelland J.D. (2011). Mental retardation and human chromosome 21 gene overdosage: From functional genomics and molecular mechanisms towards preventionand treatment of the neuropathogenesis of Down syndrome. Genomics, Proteomics, and the Nervous System.

[3] Kitamura T., Saitoh Y., Takashima N., Murayama A., Niibori Y., Ageta H., Sekiguchi M., Sugiyama H., Inokuchi K. (2009). Adult neurogenesis modulates the hippocampus-dependent period of associative fear memory. Cell.

[4] Spalding K.L., Bergmann O., Alkass K., Bernard S., Salehpour M., Huttner H.B., Boström E., Westerlund I., Vial C., Buchholz B.A. (2013). Dynamics of hippocampal neurogenesis in adult humans. Cell.

[5] Stagni F., Giacomini A., Emili M., Guidi S., Bartesaghi R. (2018). Neurogenesis impairment: An early developmental defect in Down syndrome. Free Radic. Biol. Med..

[6] Bartesaghi R., Guidi S., Ciani E. (2011). Is it possible to improve neurodevelopmental abnormalities in Down syndrome?. Rev. Neurosci..

[7] Rueda N., Florez J., Martinez-Cue C. (2012). Mouse models of Down syndrome as a tool to unravel the causes of mental disabilities. Neural Plast..

[8] Gimeno A., García-Giménez J.L., Audí L., Toran N., Andaluz P., Dasí F., Viña J., Pallardó F.V. (2014). Decreased cell proliferation and higher oxidative stress in fibroblasts from Down syndrome fetuses. Preliminary study. Biochim. Biophys. Acta-Mol. Basis Dis..

[9] Hibaoui Y., Grad I., Letourneau A., Sailani M.R., Dahoun S., Santoni F.A., Gimelli S., Guipponi M., Pelte M.F., Béna F. (2014). Modelling and rescuing neurodevelopmental defect of Down syndrome using induced pluripotent stem cells from monozygotic twins discordant for trisomy 21. EMBO Mol. Med..

[10] Kann O., Kovács R. (2007). Mitochondria and neuronal activity. Am. J. Physiol. Cell. Physiol..

[11] Xavier J.M., Rodrigues C.M.P., Solá S. (2016). Mitochondria: Major regulators of neural development. Neurosci. Rev. J. Bring Neurobiol. Neurol. Psychiatry.

[12] Valenti D., de Bari L., De Filippis B., Henrion-Caude A., Vacca R.A. (2014). Mitochondrial dysfunction as a central actor in intellectual disability-related diseases: An overview of Down syndrome, autism, Fragile X and Rett syndrome. Neurosci. Biobehav. Rev..

[13] Valenti D., de Bari L., Vigli D., Bellomo F., Lacivita E., Leopoldo M., Laviola G., Vacca R.A., De Filippis B. (2017). Stimulation of the brain serotonin receptor 7 rescues mitochondrial dysfunction in female mice from two models of Rett syndrome. Neuropharmacology.

[14] D’Antoni S., de Bari L., Valenti D., Borro M., Bonaccorso C.M., Simmaco M., Vacca R.V., Catania M.V. (2020). Aberrant mitochondrial bioenergetics in the cerebral cortex of the Fmr1 knockout mouse model of fragile X syndrome. Biol. Chem..

[15] Valenti D., Braidy N., De Rasmo D., Signorile A., Rossi L., Atanasov A.G., Volpicella M., Henrion-Caude A., Nabavi S.M., Vacca R.A. (2018). Mitochondria as pharmacological targets in Down syndrome. Free Radic. Biol. Med..

[16] Vacca R.A., Bawari S., Valenti D., Tewari D., Nabavi S.F., Shirooie S., Sah A.N., Volpicella M., Braidy N., Nabavi S.M. (2019). Down syndrome: Neurobiological alterations and therapeutic targets. Neurosci. Biobehav. Rev..

[17] Bayona-Bafaluy M.P., Garrido-Pérez N., Meade P., Iglesias E., Jiménez-Salvador I., Montoya J., Martínez-Cué C., Ruiz-Pesini E. (2021). Down syndrome is an oxidative phosphorylation disorder. Redox Biol..

[18] Valenti D., Tullo A., Caratozzolo M.F., Merafina R.S., Scartezzini P., Marra E., Vacca R.A. (2010). Impairment of F1Fo-ATPase, adenine nucleotide translocator and adenylate kinase causes mitochondrial energy deficit in human skin fibroblasts with chromosome 21 trisomy. Biochem. J..

[19] Valenti D., Manente G.A., Moro L., Marra E., Vacca R.A. (2011). Deficit of complex I activity in human skin fibroblasts with chromosome 21 trisomy and overproduction of reactive oxygen species by mitochondria: Involvement of the cAMP/PKA signaling pathway. Biochem. J..

[20] Piccoli C., Izzo A., Scrima R., Bonfiglio F., Manco R., Negri R., Quarato G., Cela O., Ripoli M., Prisco M. (2013). Chronic pro-oxidative state and mitochondrial dysfunctions are more pronounced in fibroblasts from Down syndrome foeti with congenital heart defects. Hum. Mol. Genet..

[21] Izzo A., Nitti M., Mollo N., Paladino S., Procaccini C., Faicchia D., Calì G., Genesio R., Bonfigli F., Cicatiello R. (2017). Metformin restores the mitochondrial network and reverses mitochondrial dysfunction in Down syndrome cells. Hum. Mol. Genet..

[22] Valenti D., de Bari L., de Rasmo D., Signorile A., Henrion-Caud A., Contestabile A., Vacca R.A. (2016). The polyphenols resveratrol and epigallocatechin-3-gallate restore the severe impairment of mitochondria in hippocampal progenitor cells from a Down syndrome mouse model. Biochim. Biophys. Acta.

[23] Valenti D., Rossi L., Marzulli D., Bellomo F., De Rasmo D., Signorile A., Vacca R.A. (2017). Inhibition of Drp1-mediated mitochondrial fission improves mitochondrial dynamics and bioenergetics stimulating neurogenesis in hippocampal progenitor cells from a Down syndrome mouse model. Biochim. Biophys. Acta.

[24] Scala I., Valenti D., Scotto D’Aniello V., Marino M., Riccio M.P., Bravaccio C., Vacca R.A., Strisciuglio P. (2021). Epigallocatechin-3-Gallate Plus Omega-3 Restores the Mitochondrial Complex I and FoF1-ATP Synthase Activities in PBMCs of Young Children with Down Syndrome: A Pilot Study of Safety and Efficacy. Antioxidants.

[25] Spencer J.P. (2008). Flavonoids: Modulators of brain function?. Br. J. Nutr..

[26] Williams R.J., Spencer J.P. (2012). Flavonoids, cognition, and dementia: Actions, mechanisms and potential therapeutic utility for Alzheimer disease. Free Radic. Biol. Med..

[27] Atanasov A.G., Zotchev S.B., Verena M.N., Dirsch V.M. (2021). The International Natural Product Sciences Taskforce & Supuran CT. Natural products in drug discovery: Advances and opportunities. Nat. Rev. Drug Discov..

[28] Vacca R.A., Valenti D., Caccamese S., Daglia M., Braidy N., Nabavi S.M. (2016). Plant polyphenols as natural drugs for the management of Down syndrome and related disorders. Neurosci. Biobehav. Rev..

[29] Emili M., Guidi S., Uguagliati B., Giacomini A., Bartesaghi R., Stagni F. (2020). Treatment with the flavonoid 7,8-Dihydroxyflavone: A promising strategy for a constellation of body and brain disorders. Crit. Rev. Food Sci. Nutr..

[30] Guedj F., Siegel A.E., Pennings J.L.A., Alsebaa F., Massingham L.J., Tantravahi U., Bianchi D.W. (2020). Apigenin as a Candidate Prenatal Treatment for Trisomy 21: Effects in Human Amniocytes and the Ts1Cje Mouse Model. Am. J. Hum. Genet..

[31] Stagni F., Giacomini A., Guidi S., Ciani E., Bartesaghi R. (2015). Timing of therapies for Down syndrome: The sooner, the better. Front. Behav. Neurosci..

[32] Stagni F., Giacomini A., Emili M., Trazzi S., Guidi S., Sassi M., Ciani E., Rimondini R., Bartesaghi R. (2016). Short- and long-term effects of neonatal pharmacotherapy with epigallocatechin-3-gallate on hippocampal development in the Ts65Dn mouse model of Down syndrome. Neuroscience.

[33] Stagni F., Giacomini A., Guidi S., Emili M., Uguagliati B., Salvalai M.E., Bortolotto V., Grilli M., Rimondini R., Bartesaghi R. (2017). A flavonoid agonist of the TrkB receptor for BDNF improves hippocampal neurogenesis and hippocampus-dependent memory in the Ts65Dn mouse model of DS. Exp. Neurol..

[34] Stagni F., Giacomini A., Emili M., Guidi S., Ciani E., Bartesaghi R. (2017). Epigallocatechin gallate: A useful therapy for cognitive disability in Down syndrome?. Neurogenesis.

[35] Arrázola M.S., Andraini T., Szelechowski M., Mouledous L., Arnauné-Pelloquin L., Davezac N., Belenguer P., Rampon C., Miquel M.C. (2019). Mitochondria in Developmental and Adult Neurogenesis. Neurotox. Res..

[36] Valenti D., de Bari L., De Filippis B., Ricceri L., Vacca R.A. (2014). Preservation of mitochondrial functional integrity in mitochondria isolated from small cryopreserved mouse brain areas. Anal. Biochem..

[37] Trazzi S., Mitrugno V.M., Valli E., Fuchs C., Rizzi S., Guidi S., Perini G., Bartesaghi R., Ciani E. (2011). APP-dependent up-regulation of Ptch1 underlies proliferation impairment of neural precursors in Down syndrome. Hum. Mol. Genet..

[38] Lanzillotta C., Tramutola A., Di Giacomo G., Marini F., Butterfield D.A., Di Domenico F., Perluigi M., Barone E. (2021). Insulin resistance, oxidative stress and mitochondrial defects in Ts65dn mice brain: A harmful synergistic path in Down syndrome. Free Radic. Biol. Med..

[39] Beckervordersandforth R. (2017). Mitochondrial metabolism-mediated regulation of adult neurogenesis. Brain Plast..

[40] Aylward E.H., Habbak R., Warren A.C., Pulsifer M.B., Barta P.E., Jerram M., Pearlson G.D. (1997). Cerebellar volume in adults with Down syndrome. Arch. Neurol..

[41] Guidi S., Ciani E., Bonasoni P., Santini D., Bartesaghi R. (2011). Widespread proliferation impairment and hypocellularity in the cerebellum of fetuses with Down syndrome. Brain Pathol..

[42] Lambert A.J., Brand M.D. (2009). Reactive oxygen species production by mitochondria. Methods Mol. Biol..

[43] Hroudová J., Fišar Z. (2013). Control mechanisms in mitochondrial oxidative phosphorylation. Neural Regen. Res..

[44] Austin S., St-Pierre J. (2012). PGC1α and mitochondrial metabolism—Emerging concepts and relevance in ageing and neurodegenerative disorders. J. Cell. Sci..

[45] Fernandez-Marcos P.J., Auwerx J. (2011). Regulation of PGC-1α, a nodal regulator of mitochondrial biogenesis. Am. J. Clin. Nutr..

[46] Izzo A., Mollo N., Nitti M., Paladino S., Cali G., Genesio R., Bonfiglio F., Cicatiello R., Barbato M., Sarnataro V. (2018). Mitochondrial dysfunction in Down syndrome: Molecular mechanisms and therapeutic targets. Mol. Med..

[47] Bambrick L.L., Fiskun G. (2008). Mitochondrial dysfunction in mouse trisomy 16 brain. Brain Res..

[48] Reeves R.H., Irving N.G., Moran T.H., Wohn A., Kitt C., Sisodia S.S., Schmidt C., Bronson R.T., Davisson M.T. (1995). A mouse model for Down syndrome exhibits learning and behaviour deficits. Nat Genet..

[49] Valenti D., De Rasmo D., Signorile A., Rossi L., de Bari L., Scala I., Granese B., Papa S., Vacca R.A. (2013). Epigallocatechin-3-gallate prevents oxidative phosphorylation deficit and promotes mitochondrial biogenesis in human cells from subjects with Down’s syndrome. Biochim. Biophys. Acta.

[50] Coskun P.E., Busciglio J. (2012). Oxidative stress and mitochondrial dysfunction in Down’s syndrome: Relevance to aging and dementia. Curr. Gerontol. Geriatr. Res..

[51] Schuchmann S., Heinemann U. (2000). Increased mitochondrial superoxide generation in neurons from trisomy 16 mice: A model of Down’s syndrome. Free Radic. Biol. Med..

[52] Capone G., Kim P., Jovanovich S., Payne L., Freund L., Welch K., Miller E., Trush M. (2002). Evidence for increased mitochondrial superoxide production in Down syndrome. Life Sci..

[53] Wood J., Tse M.C.L., Yang X., Brobst D., Liu Z., Pang B.P.S., Chan W.S., Zaw A.M., Chow B.K.C., Ye K. (2018). BDNF mimetic alleviates body weight gain in obese mice by enhancing mitochondrial biogenesis in skeletal muscle. Metabolism.

[54] Zhao J., Du J., Pan Y., Chen T., Zhao L., Zhu Y., Chen Y., Zheng Y., Liu Y., Sun L. (2016). Activation of cardiac TrkB receptor by its small molecular agonist 7,8-dihydroxyflavone inhibits doxorubicin-induced cardiotoxicity via enhancing mitochondrial oxidative phosphorylation. Free Radic. Biol. Med..

[55] Wang Z., Wang S., Qun Shao Q., Li P., Sun Y., Luo L., Yan X., Fan Z., Hu J., Zhaod J. (2019). Brain-derived neurotrophic factor mimetic, 7,8-dihydroxyflavone, protects against myocardial ischemia by rebalancing optic atrophy 1 processing. Free Radic. Biol. Med..

[56] Agrawal R., Noble E., Tyagi E., Zhuang Y., Ying Z., Gomez-Pinilla F. (2015). Flavonoid derivative 7,8-DHF attenuates TBI pathology via TrkB activation. Biochim. Biophys. Acta.

[57] Bryant J., Andhavarapu S., Bever C., Guda P., Katuri A., Gupta U., Arvas M., Asemu G., Heredia A., Gerzanich V. (2021). 7,8-Dihydroxyflavone improves neuropathological changes in the brain of Tg26 mice, a model for HIV-associated neurocognitive disorder. Sci. Rep..

[58] Raefsky S.M., Mattson M.P. (2017). Adaptive responses of neuronal mitochondria to bioenergetic challenges: Roles in neuroplasticity and disease resistance. Free Radic. Biol. Med..

[59] Stagni F., Uguagliati B., Emili M., Giacomini A., Bartesaghi R., Guidi S. (2021). The flavonoid 7,8-DHF fosters prenatal brain proliferation potency in a mouse model of Down syndrome. Sci. Rep..

[60] Bimonte-Nelson H.A., Hunter C.L., Nelson M.E., Granholm A.C. (2003). Frontal cortex BDNF levels correlate with working memory in an animal model of Down syndrome. Behav. Brain Res..

[61] Giacomini A., Stagni F., Emili M., Uguagliati B., Rimondini R., Bartesaghi R., Guidi S. (2019). Timing of Treatment with the Flavonoid 7,8-DHF Critically Impacts on Its Effects on Learning and Memory in the Ts65Dn Mouse. Antioxidants.

[62] Cheng A., Wan R., Yang J.L., Kamimura N., Son T.G., Ouyang X., Luo Y., Okun E., Mattson M.P. (2012). Involvement of PGC-1α in the formation and maintenance of neuronal dendritic spines. Nat. Commun..

[63] Ahuja P., Ng C.F., Pang B.P.S., Chan W.S., Tse M.C.L., Bi X., Kwan H.R., Brobst D., Herlea-Pana O., Yang X. (2021). Muscle-generated BDNF (brain derived neurotrophic factor) maintains mitochondrial quality control in female mice. Autophagy.

